# Naloxegol, an Oral Peripherally Acting Opioid Receptor Antagonist, Administered Concurrently with First-Line Systemic Therapy for Advanced Lung Adenocarcinoma (Alliance A221504): A Feasibility and Safety Study

**DOI:** 10.3390/cancers18030373

**Published:** 2026-01-25

**Authors:** Pankaj Gupta, Kalpna Gupta, Travis Dockter, Elizabeth Harlos, Selina Chow, Niveditha Subbiah, Kathryn J. Ruddy, Lyudmila Bazhenova, Shelby Terstriep, Chao H. Huang, Robert A. Kratzke, Everett E. Vokes, Charles L. Loprinzi

**Affiliations:** 1 VA Long Beach Healthcare System, Long Beach, CA 90822, USA; 2 Department of Medicine, University of California Irvine, Orange, CA 92868, USA; kalpnag@hs.uci.edu; 3 Alliance Statistics and Data Management Center, Mayo Clinic, Rochester, MN 55905, USA; dockter.travis@mayo.edu (T.D.);; 4 Alliance Protocol Operations Office, University of Chicago, Chicago, IL 60606, USA; schow@medicine.bsd.uchicago.edu (S.C.); niveditha@alliancenctn.org (N.S.); 5 Department of Medical Oncology, Mayo Clinic Comprehensive Cancer Center, Mayo Clinic, Rochester, MN 55905, USA; ruddy.kathryn@mayo.edu (K.J.R.); cloprinzi@mayo.edu (C.L.L.); 6 Department of Medicine, University of California San Diego, San Diego, CA 92093, USA; lbazhenova@health.ucsd.edu; 7 Sanford Roger Maris Cancer Center, Fargo, ND 58102, USA; shelby.terstriep@sanfordhealth.org; 8 VA Medical Center, Kansas City, MI 64128, USA; chuang2@kumc.edu; 9 University of Kansas Hospital-Westwood Cancer Center, Kansas City, MI 66205, USA; 10 Department of Medicine, University of Minnesota, Minneapolis, MN 55455, USA; kratz003@umn.edu; 11 Departments of Medicine and Radiation and Cellular Oncology, University of Chicago, Chicago, IL 60637, USA; evokes@medicine.bsd.uchicago.edu

**Keywords:** antineoplastic agents, constipation, lung neoplasms, narcotic antagonists, pain, quality of life, randomized controlled trial, receptors, opioid

## Abstract

**Background**: Mu opioid receptors (MORs) in peripheral tissues mediate adverse effects of opioids that impair health-related quality of life (HRQoL) and may stimulate cancer progression via mitogenic signaling. Naloxegol, a peripherally acting MOR antagonist (PAMORA), is approved for opioid-induced constipation. Safety and efficacy of naloxegol have not been evaluated concurrently with systemic cancer therapy. **Methods**: We conducted a randomized, double-blind, placebo-controlled trial of naloxegol in patients with advanced lung adenocarcinoma starting first-line systemic therapy. **Results**: Only 50 patients were enrolled; the trial was terminated early due to slow accrual. Two of the three components of the feasibility primary endpoint were not met (accrual and PRO completion). At 6 months, FACT-L emotional well-being was better with naloxegol (*p* = 0.0113). There were trends towards better Trial Outcome Index (*p* = 0.0505) and physical well-being (*p* = 0.0628) with naloxegol. Bowel function favored naloxegol for constipation (*p* = 0.0223), rectal pain during defecation (*p* = 0.0075), and abdominal pain from constipation (*p* = 0.0113). Adverse event frequency and severity, PRO-CTCAE, urinary hesitancy, pain scores, and progression-free and overall survival were comparable between naloxegol and placebo. **Conclusions**: Naloxegol appears to be safe and tolerable, with a signal of improved HRQoL and previously unappreciated benefit for emotional well-being, without adverse clinical outcomes. Our findings should be confirmed in larger studies. ClinicalTrials.gov ID: NCT03087708.

## Simple Summary

Opioids relieve cancer-related pain by acting on receptors in the brain. Opioids acting on receptors in peripheral tissues mediate adverse effects, e.g., constipation. Research on cells and animals suggests that opioid receptors on cancer cells and blood vessels may stimulate cancer progression. Medications (including naloxegol) that only block opioid receptors in peripheral tissues are approved to treat opioid-induced constipation. To examine the feasibility, safety, and benefits of naloxegol in patients with cancer, we conducted a trial of naloxegol vs. placebo in patients with advanced lung adenocarcinoma starting systemic therapy. Feasibility included the accrual rate, ≥grade 3 adverse events, and completion of patient-reported outcome questionnaires. The trial was closed early due to slow accrual, limiting the ability to assess endpoints. There was no undesirable impact on adverse events, patient-reported symptoms, pain, or clinical outcomes. Patients receiving naloxegol experienced better emotional well-being, constipation, and pain related to constipation, which needs confirmation in larger studies.

## 1. Introduction

The most common cause of cancer deaths is lung cancer, the second most common cancer across the world [1] that is also the third most common cancer type causing pain [2]. Opioid medications are commonly used for the treatment of severe, chronic cancer pain. Activation of mu opioid receptors (MORs) in the central nervous system (CNS) mediates opioid-induced analgesia. However, peripheral tissues also express opioid receptors (peripheral MORs), including endothelial cells [3,4,5] and human malignancies such as lung [6,7,8] and prostate [9] cancer. In patients with advanced malignancies, symptoms related to progression of cancer and its treatments, as well as the adverse effects of commonly used opioids, all contribute to impairing health-related quality of life (HRQoL) [10,11].

Compelling pre-clinical studies have indicated that expression and activation of peripheral MORs are associated with tumor progression and shorter survival in animal models and that blocking opioid receptor (OR) activity inhibits tumor growth [3,4,12]. Recent clinical studies suggest that opioid exposure may also be associated with cancer progression and shorter survival in patients with malignancies, including lung cancer [9,13,14,15,16,17,18,19,20,21]. Specifically, in lung cancer, a large number of studies have reported basic and pre-clinical data demonstrating opioid receptor (OR) expression and activity and retrospective clinical studies have shown that opioid exposure is associated with poorer clinical outcomes [6,8,13,16,20,22,23,24,25].

The hypothesis for the current study was that activation of peripheral MORs would promote cancer progression while concurrently mediating the distressing side effects of opioid analgesics such as constipation, together leading to worsening of disease-related symptoms and impairment of HRQoL. Recently, several peripherally acting mu opioid receptor antagonists (PAMORAs) have been approved for clinical use for the treatment of opioid-induced constipation (OIC) that is mediated by peripheral ORs, without compromising the analgesic effect of opioids that is mediated by MORs in the CNS [11,26,27]. The PAMORA naloxegol appears to be safe when administered to patients with cancer for treatment of opioid-induced constipation [28,29,30], but its safety and efficacy in patients receiving systemic anticancer treatment remain to be determined. We hypothesized that selective peripheral MOR inhibition may ameliorate the undesirable effects of MOR activation (e.g., tumor progression and peripheral adverse effects), without compromising centrally mediated analgesia, thereby improving HRQoL.

According to the United States Food and Drug Administration (FDA) and the American Society of Clinical Oncology (ASCO), it is important in patients with advanced NSCLC to determine the effect of treatment on symptoms and HRQoL [31,32]. Phase III trials comparing chemotherapy regimens in NSCLC have reported on HRQoL as a primary endpoint [33,34]. We therefore believed that HRQoL could be a clinically relevant measure of the potential benefit of a PAMORA that may reduce the peripheral adverse effects of opioids (e.g., constipation) as well as opioid-induced tumor progression.

In this pilot study, we examined whether long-term administration of naloxegol, an orally available, FDA-approved PAMORA, was feasible and safe in patients receiving first-line standard systemic therapy for advanced lung adenocarcinoma. In doing so, we examined the novel roles of OR antagonism using naloxegol on HRQoL, bowel function, pain, and clinical outcomes.

## 2. Materials and Methods

Naloxegol (PEGylated naloxone; Movantik^®^) is an oral PAMORA that is effective for prolonged treatment of OIC [35]. It is approved for the treatment of OIC in adults with non-cancer pain by the US FDA and for use in patients with or without cancer by the European Medicines Agency. Naloxegol is administered only once a day by mouth. It does not adversely affect analgesia. It is well tolerated: the main adverse effects are mild to moderate gastrointestinal symptoms that are transient [30,35,36,37,38,39,40]. In long-term studies (537 patients for ≥6 months and 426 patients for ≥12 months), it is safe and effective for treatment of OIC [30,37,40]. There is no risk for dependency/abuse. It is not a controlled substance [41].

The Alliance 221504 study was a randomized, double blind, placebo-controlled, three-arm pilot trial to determine the safety and feasibility of naloxegol and identify its optimal dose (from among two FDA-approved doses of 12.5 mg and 25 mg once a day) when administered concurrently with systemic therapy for advanced lung adenocarcinoma. The study was conducted by the Alliance for Clinical Trials (NCT03087708, 16 March 2017) at the Oncology National Community Oncology Research Program (NCORP) research base, and participating sites were either academic or community oncology practices. The study was conducted in accordance with the Declaration of Helsinki and was reviewed and approved by the NCI Division of Cancer Prevention (DCP) and the NCI Central Institutional Review Board. All subjects provided informed consent prior to participation in the study.

Thirty-five sites enrolled at least one patient in the study. Patients were randomized at a 1:1:1 ratio to either one of the two FDA-approved doses of naloxegol (12.5 mg or 25 mg daily by mouth) or a matching placebo. Naloxegol and matching placebo for this study were supplied by AstraZeneca (Wilmington, DE, USA) and RedHill Biopharma Ltd. (Raleigh, NC, USA). Study treatment was continued for 2 years, irrespective of cancer progression, or discontinued earlier for unacceptable adverse effects or withdrawal of consent for treatment. Two doses of naloxegol were tested to achieve the best possible balance between improving the chances of identifying possible activity, while maintaining tolerability/safety, and limiting the total sample size.

Major eligibility criteria included (i) advanced (stage IIIB/IIIC/IV) lung adenocarcinoma without known EGFR or EML4-ALK driver mutations, initiating first-line systemic therapy (chemotherapy +/− immunotherapy or +/− bevacizumab, or immunotherapy alone, at the discretion of the treating oncologist), (ii) no more than 7 days of prior use of mixed opioid agonist/opioid antagonists or other opioid antagonists within 4 weeks before registration, (iii) no methadone within 4 weeks prior to registration, (iv) age ≥ 18 years, (v) performance status Eastern Cooperative Oncology Group (ECOG) 0–2, (vi) not pregnant and not nursing, and (vii) calculated creatinine clearance ≥ 60 mL/min. Key exclusion criteria included (i) conditions that may compromise the blood–brain barrier and (ii) gastrointestinal or cardiac conditions or hepatic impairment that may increase the risk of participation. Concurrent use of opioids prior to registration or during the study was permitted as required. Detailed eligibility criteria are provided in the study protocol in the Supplemental Materials.

As detailed in Appendix A and in the study protocol, the primary endpoint was the feasibility of the study and safety of naloxegol. Secondary endpoints included HRQoL, patient-reported outcomes, OIC, pain and analgesic use, and clinical outcomes of systemic therapy.

Due to the earlier than planned termination of the trial because of slow accrual and a lack of power for the originally planned statistical tests, descriptive statistics and statistical plots formed the foundation of the statistical analyses for the secondary endpoints. Differences in HRQoL scores were assessed using Fisher’s exact test or the Wilcoxon rank sum test. Patients with missing PRO data were excluded from the HRQoL analyses. PFS was defined as the time from randomization to disease progression or death due to any cause, whichever came first. Patients who were alive and had not experienced disease progression were censored at the date of their last disease assessment. Patients who were alive with no post-baseline disease assessments were censored at the date of randomization + 1 day. OS was defined as the time from randomization to death due to any cause. Patients who were alive were censored at the date of last known contact. Kaplan–Meier curves were generated for both PFS and OS, and median (95% CI) survival times were calculated. Differences in PFS and OS were assessed using a log rank test. Hazard ratios (95% CI) exploring the effect of naloxegol compared to placebo on both PFS and OS were obtained using a univariate Cox proportional hazards model. All analyses were conducted using SAS v. 9.4.

The Alliance Statistics and Data Management Center collected data and performed statistical analyses. Data quality was reviewed by the Alliance Statistics and Data Management Center and by the study chairperson in accordance with Alliance policies. The trial was monitored at least twice annually by the Alliance Data and Safety Monitoring Board, a standing committee composed of individuals from within and outside of the Alliance. All analyses were based on the study database that was frozen on 6 October 2022.

## 3. Results

**Figure 1:**
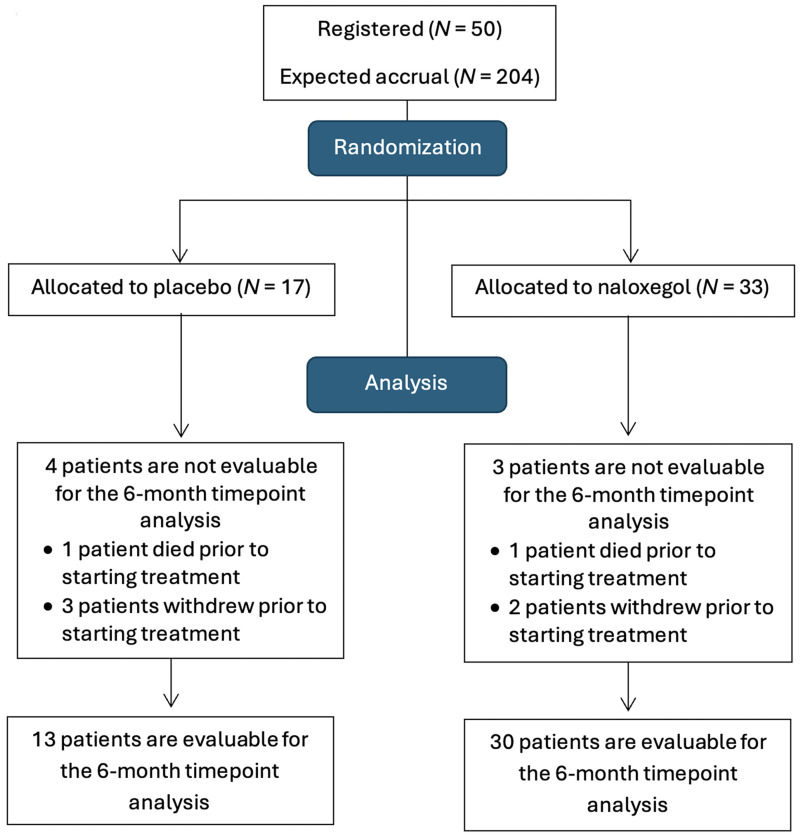
Consort Diagram.

The randomization and analysis of patients enrolled in the study are shown in Figure 1. Of 50 patients enrolled in the study, 17 were randomized to naloxegol 12.5 mg daily, 16 patients to naloxegol 25 mg daily, and 17 to placebo. Since there did not appear to be any meaningful differences in safety or tolerability of naloxegol, or in clinical outcomes between patients on the two doses of naloxegol, these two groups were combined for all analyses (*N* = 33).

This study opened on 13 October 2017, with the goal of accruing 204 patients. After 2 years and 8 months, a total of 50 (24.5% of 204) patients had been enrolled and the study was closed on 30 June 2020, without meeting the accrual goal, due to slow accrual issues. Thus, the accrual component of the primary endpoint was not met. Seven of these fifty patients were not evaluable for the 6-month timepoint analysis for the primary endpoint due to death prior to starting treatment (placebo: one patient, naloxegol: one patient) and patient withdrawal prior to starting treatment (placebo: three patients, naloxegol: two patients). Of the 43 patients evaluable for the 6-month timepoint HRQoL analysis for the primary endpoint at the time of analysis (6 October 2022), 19 (success rate: 44%, 95% CI, 29–60%) remained on treatment and alive for 6 months while completing the HRQoL booklets at each treatment cycle.

Further analysis was performed on the 43 patients (placebo: 13 and naloxegol: 30) who received at least one dose of study medication and were evaluable for the primary endpoint. Patient characteristics and stratification factors are shown in Table 1.

The study continued without meeting toxicity stopping criteria, thus the safety endpoint of the study was met. The reasons for patients coming off study treatment are shown in Table 2.

Adverse events at least possibly related to study treatment are shown in Table 3, and adverse events regardless of relationship to study treatment are shown in Supplemental Tables S1 and S2, supporting that naloxegol did not cause toxicity.

At baseline, both groups were well balanced regarding HRQoL as assessed by FACT-L scores, PRO-CTCAE, urinary hesitancy questions, and the bowel function diary, as well as in their average pain scores over the past 7 days (naloxegol: median 5.5, IQR 3.5, 8.0 vs. placebo: 4.5, IQR 4.0, 7.0; *p* = 0.7546), as shown in Supplemental Table S3a–d.

At the 6-month endpoint (Table 4 and Table 5), patients on naloxegol reported significantly better emotional well-being than those on placebo (naloxegol: median 23, IQR 21, 24 vs. placebo: 17, IQR 13, 18; *p* = 0.0113). There were trends towards a better Trial Outcome Index (naloxegol: median 65, IQR 58, 69.5 vs. placebo: 53, IQR 40, 80; *p* = 0.0505) and physical well-being (naloxegol: median 28, IQR 22.5, 28 vs. placebo: 22, IQR 15.2, 25.7; *p* = 0.0628) favoring patients receiving naloxegol. Bowel function responses favored naloxegol for constipation (naloxegol: median 5, IQR 4, 5 vs. placebo: 3, IQR 3, 4; *p* = 0.0223), rectal pain during defecation (lower is better; naloxegol: median 1, IQR 1, 1 vs. placebo: 2, IQR 2, 2; *p* = 0.0075), and abdominal pain due to constipation (lower is better; naloxegol: median 1, IQR 1, 1 vs. placebo: 3, IQR 1, 4; *p* = 0.0113). PRO-CTCAE and urinary hesitancy responses were similar in the two groups (Supplemental Table S4a). Importantly, there were no significant differences between the groups in general pain scores (naloxegol: median 2.5, IQR 0.0, 6.0 vs. placebo: 2.2, IQR 0.0, 5.7; *p* = 0.9114, Supplemental Table S4b). There were no significant differences in the requirement/use of non-opioid or opioid pain medications (Supplemental Table S5). There were also no significant differences in the change from baseline to 6 months in the FACT-L scores, urinary hesitancy questions, and average pain scores over the past 7 days (naloxegol: median −0.5, IQR −2.0, 0.0 vs. placebo: 0.2, IQR −4.5, 0.7; *p* = 0.5154), as shown in Supplemental Table S6a–d.

**Figure 2:**
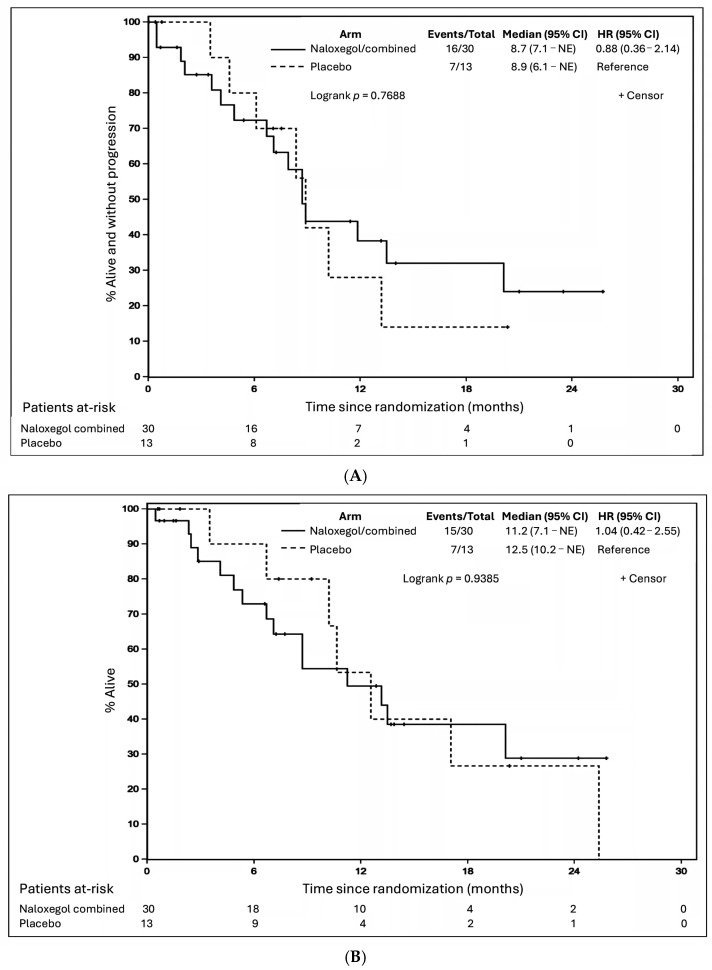
(**A**): Progression-free survival. (**B**): Overall survival.

First-line systemic cancer therapies received by patients in the two groups were statistically comparable (Supplemental Table S5). A numerically smaller proportion of patients in the naloxegol group received the most effective triplet therapy (platinum-based doublet plus immunotherapy) compared to the placebo group (17/30 (56.7%) vs. 11/13 (91.7%) patients). Nevertheless, PFS and OS were similar between the two groups (Figure 2A,B). The median PFS was 8.7 months (95% CI, 7.1-NE) and 8.9 months (95% CI, 6.1-NE) in patients on naloxegol and placebo, respectively (*p* = 0.7688). The median OS was 11.2 months (95% CI, 7.1-NE) and 12.5 months (95% CI, 10.2-NE) in patients on naloxegol and placebo, respectively (*p* = 0.9385).

## 4. Discussion

This is the first randomized, double-blind, placebo-controlled prospective study of concurrent administration of a PAMORA in patients with advanced cancer receiving systemic anticancer therapy. The main findings of this pilot study are that administration of the PAMORA naloxegol concurrently with systemic therapy for advanced lung adenocarcinoma appears to be safe and tolerable, with a signal of improved HRQoL, including a previously unappreciated benefit on emotional well-being, and without any evident adverse impact on clinical outcomes. However, despite energetic efforts by the study team, the study experienced slow enrollment due to reasons that are unknown. After careful consideration by the Alliance, the study was terminated without meeting its accrual goal. The tests performed are therefore underpowered. This could result in some differences between arms being missed because of the small sample sizes. Additionally, more patients on the placebo arm were not evaluable for the 6-month analysis, which could have biased our results. There may also have been a confounding factor on our results due to a higher proportion of current smokers in the placebo arm.

Naloxegol was well tolerated and safe in the current study, consistent with other studies that evaluated this product. In patients who already had OIC refractory to laxatives, adverse events leading to discontinuation of naloxegol occurred in 10.5% non-cancer patients [40] and in 4.8–8.7% of cancer patients [30,36,39] in short- and long-term studies. These early, primarily gastrointestinal events (diarrhea, abdominal pain, nausea/vomiting) may have been related at least partly to the restoration of bowel function in the setting of severe OIC. This possibility is supported by the finding that the discontinuation rate of naloxegol in the current study was no higher than that of placebo, probably because patients did not have OIC at study entry.

For the secondary endpoints of this study (HRQoL, patient-reported outcomes, urinary retention, constipation, pain, analgesic use, PFS, and OS), this pilot study demonstrated a signal of modest improvement for some measures in the naloxegol group and no difference from placebo in other measures. On the FACT-L instrument, administration of naloxegol was associated with significantly better emotional well-being and trends towards a better Trial Outcome Index and physical well-being. Even though high opioid use or the presence of OIC was not required for study entry, subjects receiving naloxegol reported significantly less constipation, rectal pain, and abdominal pain compared to those receiving placebo. Notably, administration of this peripherally acting MOR antagonist did not worsen pain scores.

PAMORAs were synthesized so that they had little or no effect in the CNS, while being effective in antagonizing the distressing gastrointestinal side effects of opioids. Naloxegol is a pegylated form of naloxone, which limits its ability to cross the blood–brain barrier and makes it a substrate for efflux by P-glycoprotein [42]. It functions as a competitive antagonist of MOR in peripheral tissues [43,44]. Findings in the current study were consistent with the known pharmacology of naloxegol.

Recent prospective observational single-arm studies from Europe (where naloxegol is approved for use in patients with cancer) in patients with various types of cancer reported that naloxegol improved refractory OIC and improved QoL specifically related to improvement in OIC. Davies et al. reported on a short-term (4-week) single-arm, open label study of naloxegol in patients with more than nine types of cancer (including lung cancer) with OIC, and while 46.2% patients were receiving chemotherapy, no information was provided on chemotherapy-related safety or outcomes [36]. Similarly, Lemaire et al. conducted a short-term (4-week), single-arm study of naloxegol for treatment of OIC in patients with more than seven types of malignancy, including lung cancer; 54% patients were receiving chemotherapy, but chemotherapy-related outcomes were not available [38]. Another short-term (4-week) observational single-arm study reported on patients with more than five different cancers receiving palliative care, but none of the patients was receiving chemotherapy [39]. A 3-month single-arm, observational study on patients with lung, breast, gastrointestinal, prostate, and other cancers treated with naloxegol for OIC described the improvement in OIC and related HRQoL but did not include the effect of naloxegol on the safety or outcomes of chemotherapy [29]. Finally, Cobo Dols et al. published a one-year follow-up of patients with lung, breast, gastrointestinal, prostate, and other malignancies treated with naloxegol for OIC, but information on systemic cancer treatment was not included [30]. In contrast to our trial, therefore, none of these prior studies comprised a homogenous population of patients with a single type of advanced cancer receiving systemic anticancer treatment and treated in a randomized, double-blind manner with naloxegol vs. matching placebo (irrespective of OIC) and evaluated for potential effects of naloxegol on the safety and efficacy of anticancer therapies.

A pooled post hoc analysis of 229 patients with end-stage cancer on palliative care and experiencing opioid-induced constipation enrolled in two randomized, placebo-controlled trials found that administration of methylnaltrexone was associated with longer OS [45]. However, the effect of methylnaltrexone or other PAMORAs in patients receiving systemic anticancer therapies has not been examined previously. Because of the small sample size of the current study, the 95% CI for median PFS and OS is wide. Nevertheless, there did not appear to be any obvious difference in outcomes between subjects receiving naloxegol vs. placebo. The fact that PFS and OS did not appear to be adversely impacted by naloxegol provides additional reassurance that it can probably be administered safely with systemic anticancer treatments.

## 5. Conclusions

We report the first randomized, double-blind, placebo-controlled study of a PAMORA administered concurrently with systemic cancer therapy for advanced lung cancer. Naloxegol was safe and tolerable. The limitation of a smaller than required sample size limits its effectiveness on multiple outcomes but offers a suggestion of improvement in emotional and other aspects of HRQoL, without adverse effects on clinical outcomes. Our findings may inform the design of future studies that examine the effect of PAMORAs on HRQoL in patients receiving diverse anticancer treatments for various malignancies.

## Figures and Tables

**Table 1:** Patient characteristics and stratification factors, including all patients evaluable for the 6-month primary endpoint analysis.

	Placebo (*N* = 13)	Naloxegol (*N* = 30)	Total (*N* = 43)
**Age (In Years)**			
Mean (SD)	63.2 (7.54)	66.7 (7.87)	65.6 (7.86)
Median (IQR)	62.0 (60.0, 67.0)	66.5 (62.0, 73.0)	66.0 (60.0, 71.0)
**Race**			
White	12 (92.3%)	23 (76.7%)	35 (81.4%)
Black or African American	1 (7.7%)	4 (13.3%)	5 (11.6%)
American Indian or Alaskan Native	0 (0.0%)	1 (3.3%)	1 (2.3%)
Asian	0 (0.0%)	1 (3.3%)	1 (2.3%)
Unknown: Patient unsure	0 (0.0%)	1 (3.3%)	1 (2.3%)
**Sex**			
Female	4 (30.8%)	12 (40.0%)	16 (37.2%)
Male	9 (69.2%)	18 (60.0%)	27 (62.8%)
**Ethnicity**			
Not Hispanic or Latino	12 (92.3%)	29 (96.7%)	41 (95.3%)
Hispanic or Latino	0 (0.0%)	1 (3.3%)	1 (2.3%)
Not reported	1 (7.7%)	0 (0.0%)	1 (2.3%)
**Planned use of Bevacizumab**			
No	13 (100.0%)	28 (93.3%)	41 (95.3%)
Yes	0 (0.0%)	2 (6.7%)	2 (4.7%)
**ECOG Performance Score**			
0–1	11 (84.6%)	22 (73.3%)	33 (76.7%)
2	2 (15.4%)	8 (26.7%)	10 (23.3%)
**Smoking Status**			
Current smoker	8 (61.5%)	10 (33.3%)	18 (41.9%)
Former smoker (no smoking for ≥1 year)	4 (30.8%)	19 (63.3%)	23 (53.5%)
Never smoked (<100 cigarettes in lifetime)	1 (7.7%)	1 (3.3%)	2 (4.7%)

**Table 2:** Reasons for discontinuation of study treatment, including all 50 randomized patients.

	Placebo (*N* = 17)	Naloxegol (*N* = 33)	Total (*N* = 50)
Treatment Completed Per Protocol	1 (5.9%)	3 (9.1%)	4 (8.0%)
Patient Withdrawal/Refusal After Beginning Treatment	5 (29.4%)	8 (24.2%)	13 (26.0%)
Adverse Events/Side Effects/Complications	1 (5.9%)	2 (6.1%)	3 (6.0%)
Off Treatment For Other Complicating Diseases	0 (0.0%)	1 (3.0%)	1 (2.0%)
Death On Study	4 (23.5%)	8 (24.2%)	12 (24.0%)
Other	3 (17.6%)	9 (27.3%)	12 (24.0%)
Patient Withdrawal/Refusal Prior To Beginning Treatment	3 (17.6%)	2 (6.1%)	5 (10.0%)

**Table 3:** Adverse events at least possibly related to study treatment.

Placebo vs. Naloxegol Combined	Placebo (*N* = 13)	Naloxegol Combined (*N* = 30)	Total (*N* = 43)	*p*-Value
Hematologic (grade)				0.2430 ^1^
0	13 (100.0%)	27 (90.0%)	40 (93.0%)	
1	0 (0.0%)	1 (3.3%)	1 (2.3%)	
2	0 (0.0%)	1 (3.3%)	1 (2.3%)	
3	0 (0.0%)	1 (3.3%)	1 (2.3%)	
Non-Hematologic (grade)				0.4581 ^1^
0	7 (53.8%)	14 (46.7%)	21 (48.8%)	
1	4 (30.8%)	8 (26.7%)	12 (27.9%)	
2	2 (15.4%)	4 (13.3%)	6 (14.0%)	
3	0 (0.0%)	4 (13.3%)	4 (9.3%)	

^1^ Kruskal–Wallis *p*-value.

**Table 4:** Quality of life (FACT-L) at 6 months.

	Placebo (*N* = 5)	Naloxegol (*N* = 14)	Total (*N* = 19)	*p*-Value
**Trial Outcome Index**				0.0505 ^1^
*N*	5	12	17	
Median (IQR)	53.0 (40.0, 60.0)	65.0 (58.0, 69.5)	64.0 (54.0, 66.0)	
Range	38.0, 65.0	43.0, 82.0	38.0, 82.0	
**Physical Well-Being**				0.0628 ^1^
*N*	5	12	17	
Median (IQR)	22.0 (15.2, 25.7)	28.0 (22.5, 28.0)	25.7 (21.0, 28.0)	
Range	14.0, 25.7	14.0, 28.0	14.0, 28.0	
**Social Function Well-Being**				0.1006 ^1^
*N*	5	12	17	
Median (IQR)	18.0 (15.0, 18.0)	23.0 (21.0, 26.0)	22.0 (18.0, 24.0)	
Range	8.0, 24.0	14.0, 28.0	8.0, 28.0	
**Emotional Well-Being**				0.0113 ^1^
*N*	5	13	18	
Median (IQR)	17.0 (13.0, 18.0)	23.0 (21.0, 24.0)	21.5 (17.0, 23.0)	
Range	11.0, 19.0	12.0, 24.0	11.0, 24.0	
**Functional Well-Being**				0.1250 ^1^
*N*	5	13	18	
Median (IQR)	16.0 (14.0, 18.0)	21.0 (17.0, 24.0)	19.5 (15.0, 24.0)	
Range	11.0, 22.0	13.0, 28.0	11.0, 28.0	
**Lung Cancer Subscale**				0.2747 ^1^
*N*	5	13	18	
Median (IQR)	19.0 (16.0, 21.0)	21.0 (19.0, 26.0)	20.5 (16.0, 24.0)	
Range	12.0, 24.0	15.0, 28.0	12.0, 28.0	

^1^ Wilcoxon rank sum *p*-value. Higher scores are better on all the scales.

**Table 5:** Bowel function diary at 6 months.

	Placebo (*N* = 5)	Naloxegol (*N* = 14)	Total (*N* = 19)	*p*-Value
**During this bowel movement, how would you describe the shape and consistency of your stool?**				0.7833 ^1^
Like a sausage or snake, smooth and soft	3 (60.0%)	5 (35.7%)	8 (42.1%)	
Like sausage but with cracks on its surface	0 (0.0%)	3 (21.4%)	3 (15.8%)	
Sausage-like but lumpy	2 (40.0%)	3 (21.4%)	5 (26.3%)	
Separate hard lumps, like nuts (hard to pass)	0 (0.0%)	1 (7.1%)	1 (5.3%)	
Soft blobs with clear-cut edges (passed easily)	0 (0.0%)	2 (14.3%)	2 (10.5%)	
**How much did you have to strain during this bowel movement?**				0.1219 ^2^
*N*	5	14	19	
Median (IQR)	2.0 (2.0, 3.0)	1.0 (1.0, 2.0)	2.0 (1.0, 2.0)	
Range	1.0, 4.0	1.0, 3.0	1.0, 4.0	
**During this bowel movement, how much did you feel that you were able to fully empty your bowels?**				0.0223 ^2^
*N*	5	14	19	
Median (IQR)	3.0 (3.0, 4.0)	5.0 (4.0, 5.0)	4.0 (3.0, 5.0)	
Range	3.0, 4.0	1.0, 5.0	1.0, 5.0	
**How much pain did you have around your rectum during this bowel movement?**				0.0075 ^2^
*N*	5	14	19	
Median (IQR)	2.0 (2.0, 2.0)	1.0 (1.0, 1.0)	1.0 (1.0, 2.0)	
Range	1.0, 3.0	1.0, 2.0	1.0, 3.0	
**In the past 24 h, how often were you unable to have a bowel movement even though you felt like you had to?**				0.5097 ^2^
*N*	5	14	19	
Median (IQR)	1.0 (1.0, 2.0)	1.0 (1.0, 1.0)	1.0 (1.0, 2.0)	
Range	1.0, 3.0	1.0, 3.0	1.0, 3.0	
**In the past 24 h, how much bloating did you feel because of constipation?**				0.3376 ^2^
*N*	5	14	19	
Median (IQR)	1.0 (1.0, 4.0)	1.0 (1.0, 1.0)	1.0 (1.0, 2.0)	
Range	1.0, 4.0	1.0, 4.0	1.0, 4.0	
**In the past 24 h, how much pain did you feel in your abdomen because of constipation?**				0.0113 ^2^
*N*	5	14	19	
Median (IQR)	3.0 (1.0, 4.0)	1.0 (1.0, 1.0)	1.0 (1.0, 1.0)	
Range	1.0, 4.0	1.0, 2.0	1.0, 4.0	
**In the past 24 h, how much were you bothered by gas?**				0.3966 ^2^
*N*	5	14	19	
Median (IQR)	1.0 (1.0, 3.0)	1.0 (1.0, 2.0)	1.0 (1.0, 2.0)	
Range	1.0, 3.0	1.0, 2.0	1.0, 3.0	
**In the past 24 h, how much were you bothered by a lack of appetite because of constipation?**				0.2691 ^2^
*N*	5	14	19	
Median (IQR)	1.0 (1.0, 2.0)	1.0 (1.0, 1.0)	1.0 (1.0, 1.0)	
Range	1.0, 3.0	1.0, 3.0	1.0, 3.0	

^1^ Fisher Exact *p*-value; ^2^ Wilcoxon rank sum *p*-value. Except for the question about the shape and consistency of the stools, all responses were on a 1–5 scale, with 1 being the lowest score and 5 the highest score.

## Data Availability

Data for this study were captured in iMedidata Rave. De-identified patient data may be requested from Alliance for Clinical Trials in Oncology via Datasharing@alliancenctn.org if data are not publicly available. A formal review process includes verifying the availability of data, conducting a review of any existing agreements that may have implications for the project, and ensuring that any transfer is in compliance with the IRB. The investigator will be required to sign a data release form prior to transfer.

## Supplementary Materials

The following supporting information can be downloaded at: https://www.mdpi.com/article/10.3390/cancers18030373/s1, Table S1: Adverse events regardless of relationship to study treatment. Table S2: Grade 5 adverse events, regardless of relationship to study treatment. Table S3: (a) Quality of Life (FACT-L) at Baseline; (b) PRO-CTCAE and urinary hesitancy questions at Baseline; (c) Bowel Function Diary at Baseline; (d) Pain Diary at Baseline. Table S4: (a) PRO-CTCAE and urinary hesitancy questions at 6 months; (b) Pain Diary at 6 months. Table S5: Analgesic use and first-line systemic cancer treatments. Table S6: (a) Quality of Life (FACT-L) change from baseline to 6 months; (b) Urinary Hesitancy change from baseline to 6 months; (c) Bowel Function Diary change from baseline to 6 months; (d) Pain Diary change from baseline to 6 months.

## Abbreviations

The following abbreviations are used in this manuscript:

ASCO	American Society of Clinical Oncology
CNS	Central Nervous System
DCP	Division of Cancer Prevention (of the National Cancer Institute)
ECOG	Eastern Cooperative Oncology Group
EML4-ALK	Echinoderm Microtubule-Associated Protein-Like 4-Anaplastic Lymphoma Kinase Fusion Gene
EGFR	Epidermal Growth Factor Receptor
EWB	Emotional Well-Being
FACT-G	Functional Assessment of Cancer Therapy-General
FACT-L	Functional Assessment of Cancer Therapy-Lung
FDA	Food and Drug Administration
FWB	Functional Well-Being
HRQoL	Health-Related Quality of Life
IQR	Inter-Quartile Range
LCS	Lung Cancer Subscale
MOR	Mu Opioid Receptor
NCI	National Cancer Institute
NCORP	NCI Community Oncology Research Program
NE	Not Evaluable
NSCLC	Non-Small Cell Lung Cancer
OIC	Opioid-Induced Constipation
OR	Opioid Receptor
OS	Overall Survival
PAMORA	Peripherally Acting Mu Opioid Receptor Antagonist
PFS	Progression-Free Survival
PRO-CTCAE	Patient Reported Outcomes-Common Terminology Criteria for Adverse Events
PWB	Physical Well-Being
SFWB	Social/Family Well-Being
TOI	Trial Outcome Index
95% CI	95% Confidence Interval

## Appendix A

As detailed in the study protocol, the primary endpoint was the feasibility of the study and safety of naloxegol in this setting, evaluated by the following criteria:

(i) the rate of accrual remaining ≥ 80% of the expected,
(ii) ≥80% of patients who remain alive at 6 months continuing the study medication and completing the HRQoL and other forms, for at least 6 months, and
(iii) the study continuing without meeting toxicity stopping criteria as specified below.

Accrual will be temporarily suspended to this study if at any time we observe events considered at least possibly related to study treatment (i.e., an adverse event with attribute specified as “possible”, “probable”, or “definite”) that satisfy the following:

- If 5 or more patients in the first 20 treated patients (or 25% of all patients after 20 are accrued) experienced a grade 4 or higher non-hematologic adverse event and there was a higher rate in the highest dose active treatment arm as opposed to the placebo arm.
- The study team reviewed grade 4 and 5 adverse events deemed “unrelated” or “unlikely to be related” to verify their attribution and to monitor the emergence of a previously unrecognized treatment-related adverse event.

Secondary endpoints included

(i) the change from baseline to 6 months in Trial Outcome Index (TOI), function subscales, and Lung Cancer Subscale (LCS) of the Functional Assessment of Cancer Therapy-Lung (FACT-L),
(ii) patient-reported outcomes assessed by PRO-CTCAEs and urinary retention,
(iii) opioid-induced constipation rating,
(iv) pain and analgesic use,
(v) clinical outcomes unexpected for the systemic therapy given,
(vi) progression-free survival (PFS), and
(vii) overall survival (OS).

The FACT-L [46] includes the FACT-G (version 4.0), developed by Cella et al. [47] as an overall cancer-specific HRQoL questionnaire, with a 9-item subscale concerning specific lung cancer issues. The FACT-G consists of a self-report 28-item HRQoL measure grouped into four subscales: physical well-being (PWB), social/family well-being (SFWB), emotional well-being (EWB), and functional well-being (FWB). Most items are rated on a 5-point Likert scale, from 0, “not at all” to 4, “very much”. When the Lung Cancer Subscale (LCS) is added to FACT-G, 9 additional items focus on symptoms and issues more specific to lung cancer patients. The FACT-L was scored according to the published scoring algorithm, and the following endpoints were computed at each timepoint: FACT-L (PWB + SFWB + EWB + FWB + LCS), Trial Outcome Index (TOI; PWB + FWB + LCS), PWB, SFWB, EWB, FWB, and LCS. Higher scores indicate better HRQoL. Recently, the NCI developed a patient-reported version of the CTCAE called the Patient-Reported Outcome-CTCAE (PRO-CTCAE). The PRO-CTCAE is a library of questions asking patients about specific symptomatic adverse events associated with cancer therapy. The frequency and severity of 8 PRO-CTCAE items were also assessed in this study. Urinary hesitancy was assessed using a visual analogue scale from 0–10 with 0 representing no symptom and 10 representing a symptom as bad as imaginable. Constipation and abdominal symptoms were assessed using modified versions of modules 1 and 2 of a validated bowel function diary [48]. Pain and analgesic use were assessed via a self-report daily medication/pain diary that patients were asked to complete every day at home. All of these questionnaires were to be completed every 3 weeks for the first year, then every 3 months during year 2 of study treatment.

The study was designed as a three-arm parallel group design with neither crossover nor interim analysis. The criteria for safety and feasibility were evaluated as follows:

(i) The observed accrual rate was calculated as the total number of patients accrued to the study over two years divided by 184, the total expected accrual of patients evaluable for the primary endpoint.
(ii) The proportion of patients alive at 6 months who continued the study drug and completed the HRQoL and other forms for at least 6 months was calculated by arm and compared to the anticipated success rate of 60% of all patients enrolled (the equivalent threshold for success in terms of the patients enrolled on the trial; that is, 80% of the patients remaining alive at 6 months continuing on study medication and completing the HRQoL and other forms, and the expectation that 75% of the patients enrolled on the study were alive at 6 months. Therefore, 80% × 75% = 60%).
(iii) The frequency of adverse events was summarized by arm and differences were compared between each treatment arm vs. the placebo arm using Fisher’s exact test or a Kruskal–Wallis test.
