# Supplementary material for: Naloxegol, an Oral Peripherally Acting Opioid Receptor Antagonist, Administered Concurrently with First-Line Systemic Therapy for Advanced Lung Adenocarcinoma (Alliance A221504): A Feasibility and Safety Study
Source: Cancers (Basel). 2026 Jan 25;18(3):373. doi: 10.3390/cancers18030373 (PMC12896856; doi:10.3390/cancers18030373)
Supplement: Supplementary file 1 [file cancers-18-00373-s001.zip › cancers-4059942-supplementary.pdf]

Supplemental Table S1: Adverse events regardless of relationship to study treatment.

	Placebo (N=13)	Naloxegol (*N*=30)	Total (*N*=43)	*p*-value
Hematologic (grade)				0.3985[1]
0	10 (76.9%)	19 (63.3%)	29 (67.4%)	
1	0 (0.0%)	3 (10.0%)	3 (7.0%)	
2	1 (7.7%)	1 (3.3%)	2 (4.7%)	
3	2 (15.4%)	5 (16.7%)	7 (16.3%)	
4	0 (0.0%)	2 (6.7%)	2 (4.7%)	
5	0 (0.0%)	0 (0.0%)	0 (0.0%)	
Non-Hematologic (grade)				0.4075[1]
0	3 (23.1%)	2 (6.7%)	5 (11.6%)	
1	1 (7.7%)	4 (13.3%)	5 (11.6%)	
2	3 (23.1%)	6 (20.0%)	9 (20.9%)	
3	3 (23.1%)	10 (33.3%)	13 (30.2%)	
4	0 (0%)	1 (3.3%)	1 (2.3%)	
5	3 (23.1%)	7 (23.3%)	10 (23.3%)	

[1]Kruskal Wallis *p*-value

Supplemental Table S2: Grade 5 adverse events, regardless of relationship to study treatment.

	Placebo (*N*=4)	Naloxegol (*N*=8)	Total (*N*=12)
Cardiac disorders	1 (25.0%)	0 (0.0%)	1 (8.3%)
Death NOS	1 (25.0%)	5 (62.5%)	6 (50.0%)
Dyspnea	0 (0.0%)	1 (12.5%)	1 (8.3%)
Gen disord and admin site conds	1 (25.0%)	0 (0.0%)	1 (8.3%)
Hydrocephalus	0 (0.0%)	1 (12.5%)	1 (8.3%)
Resp, thoracic, mediastinal	1 (25.0%)	0 (0.0%)	1 (8.3%)
Sudden death NOS	0 (0.0%)	1 (12.5%)	1 (8.3%)

NOS: not otherwise specified

Supplemental Table S3a: Quality of Life (FACT-L) at Baseline.

	Placebo (*N*=13)	Naloxegol Combined (*N*=29)	Total (*N*=42)	*p*-value
**Trial Outcome Index**				0.2154[1]
N	13	29	42	
Median (IQR)	42.0 (36.0, 54.0)	50.0 (42.0, 58.0)	48.7 (39.0, 57.0)	
Range	16.0, 71.0	25.0, 75.2	16.0, 75.2	
**Physical Well Being**				0.1726[1]
N	13	29	42	
Median (IQR)	14.0 (12.0, 21.0)	18.0 (14.0, 24.0)	17.0 (12.8, 23.0)	
Range	2.0, 26.0	6.0, 28.0	2.0, 28.0	
**Social Function Well Being**				0.5294[1]
N	13	29	42	
Median (IQR)	23.0 (22.0, 28.0)	24.0 (20.0, 25.7)	23.7 (21.0, 26.0)	
Range	21.0, 28.0	12.0, 28.0	12.0, 28.0	
**Emotional Well Being**				1.0000[1]
N	13	29	42	
Median (IQR)	18.0 (12.0, 21.0)	16.0 (14.0, 21.0)	17.0 (13.0, 21.0)	
Range	3.0, 24.0	3.0, 24.0	3.0, 24.0	
**Functional Well Being**				0.7642[1]
N	13	29	42	
Median (IQR)	13.0 (10.0, 16.0)	14.0 (9.3, 17.0)	13.5 (10.0, 17.0)	
Range	4.0, 23.0	5.0, 28.0	4.0, 28.0	
**Lung Cancer Subscale**				0.1556[1]
N	13	29	42	
Median (IQR)	15.0 (13.0, 17.0)	18.0 (15.0, 20.0)	17.0 (15.0, 20.0)	
Range	5.0, 27.0	8.0, 25.0	5.0, 27.0	

[1]Wilcoxon rank sum *p*-value;

Supplemental Table S3b: PRO-CTCAE and urinary hesitancy questions at Baseline.

	Placebo (*N*=13)	Naloxegol Combined (*N*=29)	Total (*N*=42)	*p*-value
**Any trouble with your ability to urinate easily? (Lower is Better)**				0.7591[1]
N	13	29	42	
Median (IQR)	0.0 (0.0, 3.0)	0.0 (0.0, 4.0)	0.0 (0.0, 4.0)	
Range	0.0, 6.0	0.0, 10.0	0.0, 10.0	
**In the last 7 days, what was the SEVERITY of your DRY MOUTH at its WORST?**				0.5247[2]
None	6 (46.2%)	9 (31.0%)	15 (35.7%)	
Mild	4 (30.8%)	7 (24.1%)	11 (26.2%)	
Moderate	2 (15.4%)	11 (37.9%)	13 (31.0%)	
Severe	0 (0.0%)	1 (3.4%)	1 (2.4%)	
Very severe	1 (7.7%)	1 (3.4%)	2 (4.8%)	
**In the last 7 days, how OFTEN did you have NAUSEA?**				0.4993[2]
Never	5 (38.5%)	15 (51.7%)	20 (47.6%)	
Rarely	3 (23.1%)	3 (10.3%)	6 (14.3%)	
Occasionally	3 (23.1%)	8 (27.6%)	11 (26.2%)	
Frequently	1 (7.7%)	3 (10.3%)	4 (9.5%)	
Almost constantly	1 (7.7%)	0 (0.0%)	1 (2.4%)	
**In the last 7 days, what was the SEVERITY of your NAUSEA at its WORST?**				0.4492[2]
None	4 (30.8%)	15 (51.7%)	19 (45.2%)	
Mild	5 (38.5%)	10 (34.5%)	15 (35.7%)	
Moderate	2 (15.4%)	1 (3.4%)	3 (7.1%)	
Severe	1 (7.7%)	2 (6.9%)	3 (7.1%)	
Very severe	1 (7.7%)	1 (3.4%)	2 (4.8%)	
**In the last 7 days, how OFTEN did you have VOMITING?**				1.0000[2]
Never	10 (76.9%)	21 (72.4%)	31 (73.8%)	
Rarely	1 (7.7%)	4 (13.8%)	5 (11.9%)	
Occasionally	1 (7.7%)	3 (10.3%)	4 (9.5%)	
Frequently	1 (7.7%)	1 (3.4%)	2 (4.8%)	
**In the last 7 days, what was the SEVERITY of your VOMITING at its WORST?**				1.0000[2]
None	10 (76.9%)	22 (75.9%)	32 (76.2%)	
Mild	1 (7.7%)	3 (10.3%)	4 (9.5%)	
Moderate	2 (15.4%)	3 (10.3%)	5 (11.9%)	
Severe	0 (0.0%)	1 (3.4%)	1 (2.4%)	

	Placebo (*N*=13)	Naloxegol Combined (*N*=29)	Total (*N*=42)	*p*-value
**In the last 7 days, how OFTEN did you have PAIN IN THE ABDOMEN (BELLY AREA)?**				0.4410[2]
Never	5 (38.5%)	16 (55.2%)	21 (50.0%)	
Rarely	2 (15.4%)	5 (17.2%)	7 (16.7%)	
Occasionally	4 (30.8%)	5 (17.2%)	9 (21.4%)	
Frequently	0 (0.0%)	2 (6.9%)	2 (4.8%)	
Almost constantly	2 (15.4%)	1 (3.4%)	3 (7.1%)	
**In the last 7 days, what was the SEVERITY of your PAIN IN THE ABDOMEN (BELLY AREA) at its WORST?**				0.3877[2]
None	5 (38.5%)	16 (55.2%)	21 (50.0%)	
Mild	2 (15.4%)	6 (20.7%)	8 (19.0%)	
Moderate	5 (38.5%)	6 (20.7%)	11 (26.2%)	
Severe	0 (0.0%)	1 (3.4%)	1 (2.4%)	
Very severe	1 (7.7%)	0 (0.0%)	1 (2.4%)	
**In the last 7 days, how much did PAIN IN THE ABDOMEN (BELLY AREA) INTERFERE with your usual or daily activities?**				0.6116[2]
Not at all	7 (53.8%)	17 (60.7%)	24 (58.5%)	
A little bit	3 (23.1%)	4 (14.3%)	7 (17.1%)	
Somewhat	2 (15.4%)	6 (21.4%)	8 (19.5%)	
Quite a bit	1 (7.7%)	0 (0.0%)	1 (2.4%)	
Very much	0 (0.0%)	1 (3.6%)	1 (2.4%)	
Missing	0	1	1	
**In the last 7 days, how OFTEN did you have LOOSE OR WATERY STOOLS (DIARRHEA)?**				0.7793[2]
Never	8 (61.5%)	19 (65.5%)	27 (64.3%)	
Rarely	4 (30.8%)	5 (17.2%)	9 (21.4%)	
Occasionally	1 (7.7%)	3 (10.3%)	4 (9.5%)	
Frequently	0 (0.0%)	2 (6.9%)	2 (4.8%)	
**In the last 7 days, did you have any INCREASED PASSING OF GAS (FLATULENCE)?**				1.0000[2]
Yes	6 (46.2%)	13 (44.8%)	19 (45.2%)	
No	7 (53.8%)	16 (55.2%)	23 (54.8%)	

**In the last 7 days, how OFTEN did you have a HEADACHE?**				0.5517[2]

	Placebo (*N*=13)	Naloxegol Combined (*N*=29)	Total (*N*=42)	*p*-value
Never	6 (46.2%)	13 (44.8%)	19 (45.2%)	
Rarely	5 (38.5%)	6 (20.7%)	11 (26.2%)	
Occasionally	2 (15.4%)	8 (27.6%)	10 (23.8%)	
Frequently	0 (0.0%)	2 (6.9%)	2 (4.8%)	
**In the last 7 days, what was the SEVERITY of your HEADACHE at its WORST?**				0.7895[2]
None	6 (46.2%)	16 (55.2%)	22 (52.4%)	
Mild	3 (23.1%)	5 (17.2%)	8 (19.0%)	
Moderate	4 (30.8%)	6 (20.7%)	10 (23.8%)	
Severe	0 (0.0%)	2 (6.9%)	2 (4.8%)	
**In the last 7 days, how much did your HEADACHE INTERFERE with your usual or daily activities?**				0.7542[2]
Not at all	9 (69.2%)	21 (72.4%)	30 (71.4%)	
A little bit	2 (15.4%)	4 (13.8%)	6 (14.3%)	
Somewhat	2 (15.4%)	2 (6.9%)	4 (9.5%)	
Very much	0 (0.0%)	2 (6.9%)	2 (4.8%)	
**In the last 7 days, how OFTEN did you have UNEXPECTED OR EXCESSIVE SWEATING DURING THE DAY OR NIGHTIME (NOT RELATED TO HOT FLASHES)?**				0.2490[2]
Never	6 (46.2%)	15 (51.7%)	21 (50.0%)	
Rarely	2 (15.4%)	6 (20.7%)	8 (19.0%)	
Occasionally	4 (30.8%)	2 (6.9%)	6 (14.3%)	
Frequently	1 (7.7%)	6 (20.7%)	7 (16.7%)	
**In the last 7 days, what was the SEVERITY of your UNEXPECTED OR EXCESSIVE SWEATING DURING THE DAY OR NIGHTIME (NOT RELATED TO HOT FLASHES) at its WORST?**				0.5182[2]
None	7 (53.8%)	15 (51.7%)	22 (52.4%)	
Mild	1 (7.7%)	5 (17.2%)	6 (14.3%)	
Moderate	4 (30.8%)	4 (13.8%)	8 (19.0%)	
Severe	1 (7.7%)	5 (17.2%)	6 (14.3%)	

[1]Wilcoxon rank sum *p*-value; [2]Fisher Exact *p*-value

Supplemental Table S3c: Bowel Function Diary at Baseline.

	Placebo (*N*=13)	Naloxegol Combined (*N*=29)	Total (*N*=42)	*p*-value
**During this bowel movement, how would you describe the shape and consistency of your stool?**				0.6308[1]
Fluffy pieces with ragged edges, a mushy stool	1 (8.3%)	1 (3.6%)	2 (5.0%)	
Like a sausage or snake, smooth and soft	5 (41.7%)	8 (28.6%)	13 (32.5%)	
Like sausage but with cracks on its surface	1 (8.3%)	9 (32.1%)	10 (25.0%)	
Sausage-like but lumpy	2 (16.7%)	3 (10.7%)	5 (12.5%)	
Separate hard lumps, like nuts (hard to pass)	3 (25.0%)	5 (17.9%)	8 (20.0%)	
Soft blobs with clear-cut edges (passed easily)	0 (0.0%)	1 (3.6%)	1 (2.5%)	
Watery, no solid pieces (entirely liquid)	0 (0.0%)	1 (3.6%)	1 (2.5%)	
Missing	1	1	2	
**How much did you have to strain during this bowel movement?**				0.2630[2]
N	12	28	40	
Median (IQR)	2.0 (1.0, 4.0)	1.0 (1.0, 3.0)	2.0 (1.0, 3.0)	
Range	1.0, 4.0	1.0, 5.0	1.0, 5.0	
**During this bowel movement, how much did you feel that you were able to fully empty your bowels?**				0.6548[2]
N	12	28	40	
Median (IQR)	3.5 (2.5, 5.0)	4.0 (3.0, 5.0)	4.0 (3.0, 5.0)	
Range	2.0, 5.0	1.0, 5.0	1.0, 5.0	
**How much pain did you have around your rectum during this bowel movement?**				0.2822[2]
N	12	29	41	
Median (IQR)	1.0 (1.0, 2.0)	1.0 (1.0, 1.0)	1.0 (1.0, 2.0)	
Range	1.0, 3.0	1.0, 5.0	1.0, 5.0	
**In the past 24 hours, how often were you unable to have a bowel movement even though you felt like you had to?**				0.4310[2]
N	13	28	41	
Median (IQR)	1.0 (1.0, 2.0)	1.0 (1.0, 3.5)	1.0 (1.0, 2.0)	
Range	1.0, 3.0	1.0, 5.0	1.0, 5.0	

	Placebo (*N*=13)	Naloxegol Combined (*N*=29)	Total (*N*=42)	*p*-value
**In the past 24 hours, how much bloating did you feel because of constipation?**				0.3647[2]
N	13	29	42	
Median (IQR)	2.0 (1.0, 3.0)	1.0 (1.0, 3.0)	1.0 (1.0, 3.0)	
Range	1.0, 4.0	1.0, 5.0	1.0, 5.0	
**In the past 24 hours, how much pain did you feel in your abdomen because of constipation?**				0.2618[2]
N	13	28	41	
Median (IQR)	1.0 (1.0, 3.0)	1.0 (1.0, 1.5)	1.0 (1.0, 2.0)	
Range	1.0, 4.0	1.0, 5.0	1.0, 5.0	
**In the past 24 hours, how much were you bothered by gas?**				0.4313[2]
N	13	29	42	
Median (IQR)	2.0 (1.0, 3.0)	2.0 (1.0, 2.0)	2.0 (1.0, 2.0)	
Range	1.0, 4.0	1.0, 4.0	1.0, 4.0	
**In the past 24 hours, how much were you bothered by a lack of appetite because of constipation?**				0.7708[2]
N	13	29	42	
Median (IQR)	1.0 (1.0, 2.0)	1.0 (1.0, 2.0)	1.0 (1.0, 2.0)	
Range	1.0, 4.0	1.0, 4.0	1.0, 4.0	

[1]Fisher Exact *p*-value; [2]Wilcoxon rank sum *p*-value

Supplemental Table S3d: Pain Diary at Baseline.

	Placebo (*N*=12)	Naloxegol Combined (*N*=28)	Total (*N*=40)	*p*-value
**Average Pain**				0.7546[1]
N	12	28	40	
Median (IQR)	4.5 (4.0, 7.0)	5.5 (3.5, 8.0)	5.0 (4.0, 8.0)	
Range	0.0, 10.0	0.0, 9.0	0.0, 10.0	

[1]Wilcoxon rank sum *p*-value

Supplemental Table S4a: PRO-CTCAE and urinary hesitancy questions at 6 months.

	Placebo (*N*=5)	Naloxegol (*N*=14)	Total (*N*=19)	*P*-value
**Any trouble with your ability to urinate easily? (Lower is Better)**				0.5907[1]
N	5	14	19	
Median (IQR)	0.0 (0.0, 1.0)	0.0 (0.0, 0.0)	0.0 (0.0, 1.0)	
Range	0.0, 5.0	0.0, 7.0	0.0, 7.0	
**In the last 7 days, what was the SEVERITY of your DRY MOUTH at its WORST?**				0.7124[2]
None	2 (50.0%)	10 (71.4%)	12 (66.7%)	
Mild	1 (25.0%)	3 (21.4%)	4 (22.2%)	
Moderate	1 (25.0%)	1 (7.1%)	2 (11.1%)	
Missing	1	0	1	
**In the last 7 days, how OFTEN did you have a HEADACHE?**				0.2337[2]
Never	2 (40.0%)	10 (71.4%)	12 (63.2%)	
Rarely	2 (40.0%)	4 (28.6%)	6 (31.6%)	
Occasionally	1 (20.0%)	0 (0.0%)	1 (5.3%)	
**In the last 7 days, what was the SEVERITY of your HEADACHE at its WORST?**				0.1760[2]
None	2 (40.0%)	11 (78.6%)	13 (68.4%)	
Mild	1 (20.0%)	1 (7.1%)	2 (10.5%)	
Moderate	2 (40.0%)	2 (14.3%)	4 (21.1%)	
**In the last 7 days, how much did your HEADACHE INTERFERE with your usual or daily activities?**				0.2722[2]
Not at all	3 (60.0%)	12 (85.7%)	15 (78.9%)	
A little bit	2 (40.0%)	2 (14.3%)	4 (21.1%)	
**In the last 7 days, did you have any INCREASED PASSING OF GAS (FLATULENCE)?**				0.2722[2]
Yes	2 (40.0%)	2 (14.3%)	4 (21.1%)	
No	3 (60.0%)	12 (85.7%)	15 (78.9%)	
**In the last 7 days, how OFTEN did you have NAUSEA?**				0.4859[2]
Never	3 (75.0%)	10 (71.4%)	13 (72.2%)	
Rarely	0 (0.0%)	3 (21.4%)	3 (16.7%)	
Occasionally	1 (25.0%)	0 (0.0%)	1 (5.6%)	
Frequently	0 (0.0%)	1 (7.1%)	1 (5.6%)	
Missing	1	0	1	
**In the last 7 days, what was the SEVERITY of your NAUSEA at its WORST?**				0.7663[2]
None	3 (75.0%)	10 (71.4%)	13 (72.2%)	
Mild	0 (0.0%)	2 (14.3%)	2 (11.1%)	
Moderate	1 (25.0%)	1 (7.1%)	2 (11.1%)	

	Placebo (*N*=5)	Naloxegol (*N*=14)	Total (*N*=19)	*P*-value
Severe	0 (0.0%)	1 (7.1%)	1 (5.6%)	
Missing	1	0	1	
**In the last 7 days, how OFTEN did you have PAIN IN THE ABDOMEN (BELLY AREA)?**				0.1971[2]
Never	2 (50.0%)	12 (85.7%)	14 (77.8%)	
Rarely	1 (25.0%)	0 (0.0%)	1 (5.6%)	
Occasionally	1 (25.0%)	2 (14.3%)	3 (16.7%)	
Missing	1	0	1	
**In the last 7 days, what was the SEVERITY of your PAIN IN THE ABDOMEN (BELLY AREA) at its WORST?**				0.1971[2]
None	2 (50.0%)	12 (85.7%)	14 (77.8%)	
Moderate	1 (25.0%)	2 (14.3%)	3 (16.7%)	
Mild	1 (25.0%)	0 (0.0%)	1 (5.6%)	
Missing	1	0	1	
**In the last 7 days, how much did PAIN IN THE ABDOMEN (BELLY AREA) INTERFERE with your usual or daily activities?**				0.4052[2]
Not at all	3 (75.0%)	13 (92.9%)	16 (88.9%)	
A little bit	1 (25.0%)	0 (0.0%)	1 (5.6%)	
Somewhat	0 (0.0%)	1 (7.1%)	1 (5.6%)	
Missing	1	0	1	
**In the last 7 days, how OFTEN did you have UNEXPECTED OR EXCESSIVE SWEATING DURING THE DAY OR NIGHTIME (NOT RELATED TO HOT FLASHES)?**				0.7872[2]
Never	2 (40.0%)	9 (64.3%)	11 (57.9%)	
Rarely	2 (40.0%)	3 (21.4%)	5 (26.3%)	
Occasionally	1 (20.0%)	2 (14.3%)	3 (15.8%)	
**In the last 7 days, what was the SEVERITY of your UNEXPECTED OR EXCESSIVE SWEATING DURING THE DAY OR NIGHTIME (NOT RELATED TO HOT FLASHES) at its WORST?**				0.3615[2]
None	2 (40.0%)	10 (71.4%)	12 (63.2%)	
Mild	2 (40.0%)	2 (14.3%)	4 (21.1%)	
Moderate	1 (20.0%)	2 (14.3%)	3 (15.8%)	
**In the last 7 days, how OFTEN did you have VOMITING?**				1.0000[2]
Never	4 (100.0%)	11 (78.6%)	15 (83.3%)	
Rarely	0 (0.0%)	2 (14.3%)	2 (11.1%)	
Frequently	0 (0.0%)	1 (7.1%)	1 (5.6%)	

	Placebo (*N*=5)	Naloxegol (*N*=14)	Total (*N*=19)	*P*-value
Missing	1	0	1	
**In the last 7 days, what was the SEVERITY of your VOMITING at its WORST?**				1.0000[2]
None	4 (100.0%)	11 (78.6%)	15 (83.3%)	
Mild	0 (0.0%)	2 (14.3%)	2 (11.1%)	
Severe	0 (0.0%)	1 (7.1%)	1 (5.6%)	
Missing	1	0	1	

[1]Wilcoxon rank sum *p*-value; [2]Fisher Exact *p*-value;

Supplemental Table S4b: Pain Diary at 6 months.

	Placebo (*N*=5)	Naloxegol Combined (*N*=14)	Total (*N*=19)	*p*-value
**Average Pain**				0.9114[1]
N	4	14	18	
Median (IQR)	2.2 (0.0, 5.7)	2.5 (0.0, 6.0)	2.5 (0.0, 6.0)	
Range	0.0, 7.0	0.0, 9.0	0.0, 9.0	

[1]Wilcoxon rank sum *p*-value;

Supplemental Table S5: Analgesic use and first-line systemic cancer treatments.

	Placebo (*N*=13)	Naloxegol (*N*=30)	Total (*N*=43)	*p*-value
**Number of medications taken**				0.2048[1]
0	1 (8.3%)	6 (20.7%)	7 (17.1%)	
1	6 (50.0%)	15 (51.7%)	21 (51.2%)	
2	2 (16.7%)	7 (24.1%)	9 (22.0%)	
3	3 (25.0%)	1 (3.4%)	4 (9.7%)	
Missing	1	1	2	
**Types of medications taken**				0.3573[1]
None	1 (8.3 %)	6 (20.7%)	7 (17.1%)	
Opioids-only	9 (75.0%)	20 (69.0%)	29 (70.7%)	
Analgesic-only	0 (0.0%)	2 (6.9%)	2 (4.9%)	
Combination	2 (16.7%)	1 (3.4%)	3 (7.3%)	
Missing	1	1	2	
**Oral morphine equivalent (OME)**				0.7053[2]
N	11	21	32	
Mean (SD)	148.6 (286.88)	58.4 (39.56)	89.4 (171.63)	
Median (IQR)	60 (30,90)	60 (30,90)	60 (30, 90)	
**First Line Cancer Therapy, n (%)**				0.1911[1]
Platinum Doublet + Immunotherapy (triplet)	11 (91.7%)	17 (56.7%)	28 (66.7%)	
Platinum Doublet	1 (8.3%)	9 (30.0%)	10 (23.8%)	
Immunotherapy Alone	0 (0.0%)	3 (10.0%)	3 (7.1%)	
Platinum Doublet + Bevacizumab	0 (0.0%)	1 (3.3%)	1 (2.4%)	
Missing	1	0	1	

[1]Fisher Exact *p*-value; [2]Wilcoxon rank sum *p*-value

Supplemental Table S6a: Quality of Life (FACT-L) change from baseline to 6 months.

	Placebo (*N*=5)	Naloxegol Combined (*N*=14)	Total (*N*=19)	*p*-value
**Trial Outcome Index**				0.5621[1]
N	5	12	17	
Median (IQR)	9.0 (-1.0, 10.0)	16.6 (-2.0, 20.7)	13.0 (-1.0, 20.4)	
Range	-2.0, 23.0	-23.0, 28.7	-23.0, 28.7	
**Physical Well Being**				0.0707[1]
N	5	12	17	
Median (IQR)	-0.3 (-5.8, 0.0)	3.0 (0.0, 4.1)	0.0 (-0.3, 3.0)	
Range	-7.0, 1.7	-9.0, 8.8	-9.0, 8.8	
**Social Function Well Being**				0.6346[1]
N	5	12	17	
Median (IQR)	-2.0 (-3.0, 1.0)	5.0 (-2.5, 8.0)	1.0 (-3.0, 7.0)	
Range	-6.0, 13.0	-11.0, 10.7	-11.0, 13.0	
**Emotional Well Being**				0.3220[1]
N	5	13	18	
Median (IQR)	0.0 (0.0, 1.0)	3.0 (1.0, 6.0)	2.0 (0.0, 6.0)	
Range	-5.0, 8.0	-12.0, 8.0	-12.0, 8.0	
**Functional Well Being**				0.9606[1]
N	5	13	18	
Median (IQR)	4.0 (3.0, 6.0)	6.0 (2.0, 8.0)	5.5 (2.0, 8.0)	
Range	1.0, 14.0	-9.0, 14.0	-9.0, 14.0	
**Lung Cancer Subscale**				0.6558[1]
N	5	13	18	
Median (IQR)	1.0 (-2.0, 6.0)	2.0 (0.0, 8.4)	1.5 (-2.0, 6.0)	
Range	-3.0, 6.0	-5.0, 15.0	-5.0, 15.0	

[1]Wilcoxon rank sum *p*-value

Supplemental Table S6b: Urinary Hesitancy change from baseline to 6 months.

	Placebo (*N*=5)	Naloxegol Combined (*N*=14)	Total (*N*=19)	*p*-value
**Any trouble with your ability to urinate easily?**				0.5563[1]
N	5	14	19	
Median (IQR)	0.0 (-1.0, 0.0)	0.0 (0.0, 0.0)	0.0 (0.0, 0.0)	
Range	-4.0, 1.0	-10.0, 7.0	-10.0, 7.0	

[1]Wilcoxon rank sum *p*-value;

Supplemental Table S6c: Bowel Function Diary change from baseline to 6 months.

Bowel Function Diary Change from Baseline to 6 Months (Lower is Better)
Placebo vs. Naloxegol Combined.

	Arm			
	Placebo (*N*=5)	Naloxegol Combined (*N*=14)	Total (*N*=19)	*p*-value
**How much did you have to strain during this bowel movement?**				0.1922[1]
N	5	14	19	
Median (IQR)	-1.0 (-1.0, 0.0)	0.0 (-1.0, 1.0)	0.0 (-1.0, 0.0)	
Range	-3.0, 0.0	-2.0, 2.0	-3.0, 2.0	
**During this bowel movement how much did you feel that you were able to fully empty your bowels?**				0.8868[1]
N	5	14	19	
Median (IQR)	1.0 (0.0, 1.0)	0.0 (-1.0, 2.0)	0.0 (-1.0, 2.0)	
Range	-2.0, 2.0	-2.0, 2.0	-2.0, 2.0	
**How much pain did you have around your rectum during this bowel movement?**				0.9141[1]
N	5	14	19	
Median (IQR)	0.0 (-1.0, 1.0)	0.0 (0.0, 0.0)	0.0 (0.0, 0.0)	
Range	-1.0, 1.0	-1.0, 1.0	-1.0, 1.0	
**In the past 24 hours how often were you unable to have a bowel movement even though you felt like you had to?**				0.8803[1]
N	5	14	19	
Median (IQR)	0.0 (0.0, 0.0)	0.0 (-1.0, 0.0)	0.0 (-1.0, 0.0)	
Range	-1.0, 1.0	-4.0, 2.0	-4.0, 2.0	
**In the past 24 hours how much bloating did you feel because of constipation?**				0.1807[1]
N	5	14	19	
Median (IQR)	0.0 (-1.0, 0.0)	0.0 (0.0, 0.0)	0.0 (0.0, 0.0)	
Range	-2.0, 0.0	-3.0, 3.0	-3.0, 3.0	
**In the past 24 hours how much pain did you feel in your abdomen because of constipation?**				0.3813[1]
N	5	14	19	
Median (IQR)	0.0 (0.0, 0.0)	0.0 (0.0, 0.0)	0.0 (0.0, 0.0)	
Range	0.0, 1.0	-3.0, 1.0	-3.0, 1.0	

Bowel Function Diary Change from Baseline to 6 Months (Lower is Better)
Placebo vs. Naloxegol Combined.

	Arm			
	Placebo (*N*=5)	Naloxegol Combined (*N*=14)	Total (*N*=19)	*p*-value
**In the past 24 hours how much were you bothered by gas?**				0.4393[1]
N	5	14	19	
Median (IQR)	-1.0 (-1.0, 0.0)	0.0 (-1.0, 0.0)	0.0 (-1.0, 0.0)	
Range	-1.0, 0.0	-2.0, 1.0	-2.0, 1.0	
**In the past 24 hours how much were you bothered by a lack of appetite because of constipation?**				0.6054[1]
N	5	14	19	
Median (IQR)	0.0 (-1.0, 0.0)	0.0 (0.0, 0.0)	0.0 (-1.0, 0.0)	
Range	-3.0, 1.0	-2.0, 2.0	-3.0, 2.0	

[1]Wilcoxon rank sum *p*-value

Supplemental Table S6d: Pain Diary change from baseline to 6 months.

	Placebo (*N*=5)	Naloxegol Combined (*N*=14)	Total (*N*=19)	*p*-value
**Average Pain**				0.5154[1]
N	4	14	18	
Median (IQR)	0.2 (-4.5, 0.7)	-0.5 (-2.0, 0.0)	0.0 (-2.0, 0.0)	
Range	-9.0, 1.0	-9.0, 3.7	-9.0, 3.7	

[1]Wilcoxon rank sum *p*-value.
